# Global longitudinal strain in long-term risk prediction after acute coronary syndrome: an investigation of added prognostic value to ejection fraction

**DOI:** 10.1007/s00392-024-02439-w

**Published:** 2024-03-25

**Authors:** Joel Lenell, Bertil Lindahl, David Erlinge, Tomas Jernberg, Jonas Spaak, Tomasz Baron

**Affiliations:** 1 Department of Medical Sciences, Uppsala University, Uppsala, Sweden; 2 Uppsala Clinical Research Center, Uppsala University, Uppsala, Sweden; 3 Department of Clinical Sciences, Lund University, Skåne University Hospital, Lund, Sweden; 4 Division of Cardiovascular Medicine, Dept. of Clinical Sciences, Danderyd Hospital, Karolinska Institutet, Stockholm, Sweden

**Keywords:** LVEF, GLS, ACS, Myocardial infarction, SWEDEHEART, Prognosis

## Abstract

**Aims:**

This study aimed to investigate the additional value of global longitudinal strain (GLS) on top of left ventricular ejection fraction (LVEF) in long-term risk prediction of combined death and heart failure (HF) re-hospitalization after acute coronary syndrome (ACS).

**Method and results:**

This retrospective study included patients admitted with ACS between 2008 and 2014 from the three participating university hospitals. LVEF and GLS were assessed at a core lab from images acquired during the index hospital stay. Their prognostic value was studied with the Cox proportional hazards model (median follow-up 6.2 years). A nested model comparison was performed with C-statistics.

A total of 941 patients qualified for multivariable analysis after multiple imputation of missing baseline covariables. The combined outcome was reached in 17.7% of the cases. Both GLS and LVEF were independent predictors of the combined outcome, hazard ratio (HR) 1.068 (95% CI 1.017–1.121) and HR 0.980 (95% CI 0.962–0.998), respectively. The C-statistic increased from 0.742 (95% CI 0.702–0.783) to 0.749 (95% CI 0.709–0.789) (*P* = 0.693) when GLS entered the model with clinical data and LVEF.

**Conclusion:**

GLS emerged as an independent long-term risk predictor of all-cause death and HF re-hospitalization. However, there was no significant incremental predictive value of GLS when LVEF was already known.

**Graphical Abstract:**

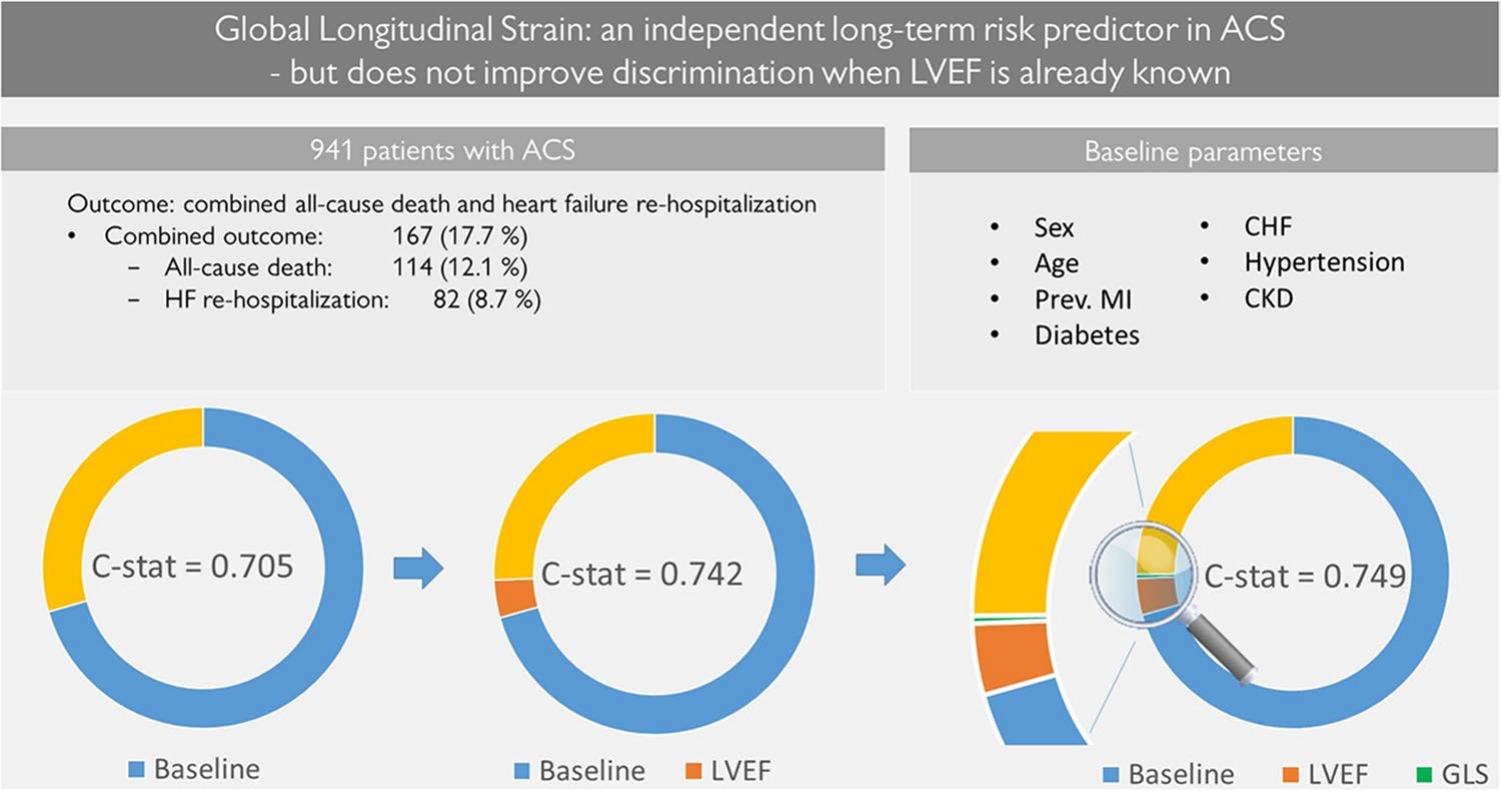

**Supplementary Information:**

The online version contains supplementary material available at 10.1007/s00392-024-02439-w.

## Introduction

Global longitudinal strain (GLS) by speckle tracking echocardiography is a sensitive marker of global systolic left ventricular (LV) function [1]. The bulk of literature in support of GLS as a predictor of adverse outcome in heart disease has grown rapidly in previous years; however, many of the studies have focused on non-ischemic heart diseases such as hypertrophic cardiomyopathy, amyloidosis, and cardiotoxic complications in oncologic treatment [2].

There is a growing interest in GLS as a prognostic marker also in ischemic heart disease and results are promising [3]. GLS has been reported to be both a predictor of LV remodeling and of left ventricular ejection fraction (LVEF) recovery after myocardial infarction (MI) [4, 5]. Outcome prediction has appeared to be particularly strong in anterior infarctions [6]. Despite GLS’s superior sensitivity in identifying myocardial dysfunction in a range of heart diseases, the dominating measurement of global LV function after MI is still LVEF [2]. This can in part be attributed to the substantial literature on LVEF as a predictor and its specific cut-off values directing clinical management.

During the last decades, there has been an improvement in LVEF among survivors of acute coronary syndrome (ACS) and an increasing number of patients are discharged with an ejection fraction around or above 50% [7]. Despite the prognostic strength of LVEF, it remains a poor predictor of outcome in subjects with ejection fraction above 40% [8]. Short- to mid-term follow-up studies have shown promising results with GLS as a more sensitive prognostic marker in this subgroup. A recent study has even found long-term predictive properties of GLS in ACS [3]. In the current study, we set out to assess whether GLS provides any clinically meaningful added prognostic value on top of clinical data and LVEF in a long-term follow-up setting.

## Methods

### Study population

This study is part of the TOTAL-AMI (Tailoring Of Treatment in All comers with Acute Myocardial Infarction) project, previously described by Eggers et al. [9].

The original population of 1385 patients was a subset from the larger TOTAL-AMI cohort with subjects hospitalized due to ACS between March 2008 and September 2014 at the departments of cardiology in Uppsala (Uppsala University Hospital, site 1), Lund (Skåne University Hospital, site 2), and Stockholm (Danderyd Hospital, site 3). This subset had been randomly singled out for multimarker panel sampling at admission and was deemed an appropriate population for this study with testing of the echocardiographic measurements given its sample size.

The subjects received guideline-directed therapy and underwent transthoracic echocardiography according to clinical routine. Medical history and patient characteristics upon presentation were retrieved from the SWEDEHEART registry. The outcome measures, time to all-cause death and time to HF re-hospitalization, were collected from the Swedish Patient Registry (PAR) in July 2018. In PAR, discharge diagnoses were based on International Classification of Diseases, 10th revision, Clinical Modification (ICD-10-CM) codes.

**Fig. 1:**
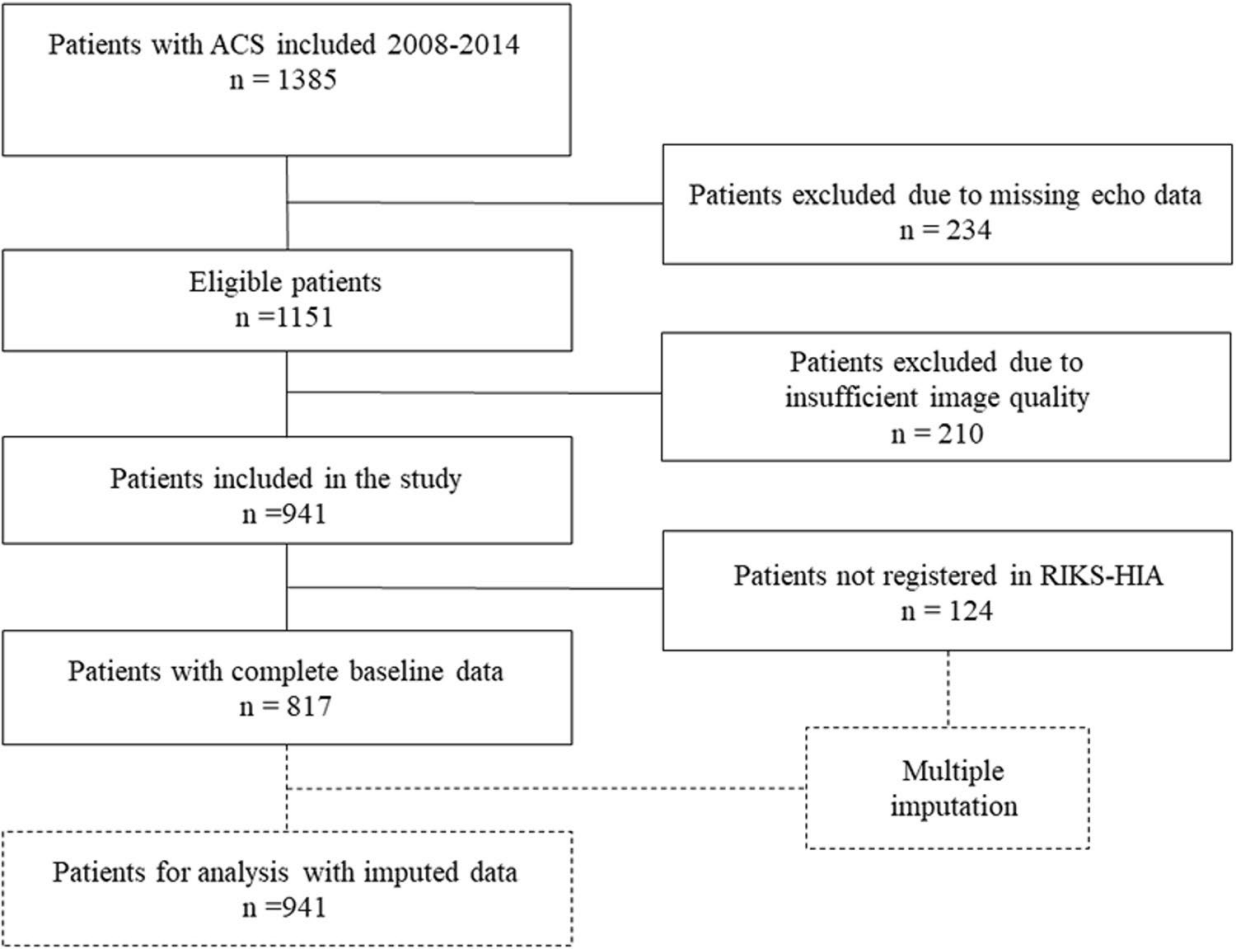
Inclusion flow chart

After exclusion of patients with missing or unsatisfactory echocardiographic images, 941 patients remained. Data on previous myocardial infarction and smoking status in the SWEDEHEART associated registry RIKS-HIA was missing in 124 patients. Missing variables were imputed by multiple imputation in the regression analyses. The inclusion process is described in Fig. 1.

The study was approved by the Regional Committee for Medical Research Ethics (DNR 2017/759–31).

### Echocardiography

Transthoracic 2D echocardiography was performed as part of clinical routine within 72 h from admission. For the purpose of this study, the echocardiographic raw data was collected from the imaging databases at the participating hospitals and re-analyzed at the core lab in Uppsala. Echocardiographic analyses were performed using TomTec-Arena version 2.30 (TomTec, Unterschleißheim, Germany). Volumetric measurements, including LVEF, were obtained by manual delineation of the endocardium and calculated according to the modified Simpson’s method. Strain measurements based on speckle tracking were obtained by software-automated delineation of the endocardium with manual corrections performed when deemed necessary. The same experienced reviewer performed all measurements.

Peak GLS was assessed in monoplane from the four-chamber-, two-chamber, and three-chamber view, respectively. Hence, triplane GLS was calculated as an average of the three views. In subjects that lacked a feasible three-chamber view, GLS was calculated as an average from the four- and two-chamber views (i.e., biplane). Patients recruited from the site in Lund were often examined according to a truncated protocol focused on biplane LVEF assessments and therefore did not always have an available apical three-chamber view (biplane GLS: *n* = 256).

In accordance with recommendations from the American Society of Echocardiography and the European Association of Cardiovascular Imaging, images with suboptimal tracking of the endocardium in more than two segments in one single view were excluded [10]. This principle was applied both in GLS and volumetric tracings. Thirteen patients (1.4%) were in atrial fibrillation during examination. Their heart rate was below 90 beats per minute and care was taken to measure GLS at somewhat regular RR intervals.

### Statistics

The predictive value of GLS and LVEF was investigated with a univariable receiver operating characteristic (ROC) analysis against the combined outcome. Their optimal cut-off values were obtained according the Youden index. Spearman’s rho was assessed to test correlation between the two echocardiographic parameters and cubic spline analyses were performed to test for a non-linear predictive relation between the echo measurements and the combined endpoint.

Clinical variables with assumed prognostic importance were selected (Table 2) for univariable analysis by Cox proportional hazards regression (congestive heart failure, hypertension, previous MI, chronic kidney disease, diabetes, smoking status). A following multivariable analysis was adjusted for clinical variables with a *p* value < 0.1 from the univariable analysis. Age and sex were included in the multivariable analysis as pre-specified covariables. Reported results from the multivariable analyses with Cox proportional hazards regression are based on imputed datasets.

Harrell’s C-index was assessed in a step-wise manner with the initial addition of LVEF to clinical data in a first model followed by GLS on top of LVEF and clinical data in a second model to evaluate improvement in model prediction. A sub-analysis of Harrell’s C was performed in the population with ejection fraction above 40% by the same step-wise approach as in the full population. The cut-off, 40%, was selected due to its clinical relevance in prognostication and treatment guidance. Change in C-index between the models within Figs. 4 and 5 was tested with DeLong’s test [11].

Differences between included and excluded patients is reported in Supplemental Table 1. An in-depth multivariable subgroup analysis with stratification for infarction type (STEMI vs NSTEMI), sex, age (cut-off at 65 years), and LVEF (cut-off at 40% and 50%) was also performed and is reported in Supplemental Table 2.

In order to explore the impact of additional baseline clinical parameters, yet with care taken as to avoid model over-fit, another stepwise analysis was performed with baseline clinical parameters of significance in ACS that were not selected for the main analysis (NTproBNP, pathologic Q-wave, bundle branch block, and infarction type). These results are presented in Supplemental Table 3. Sex stratified baseline data is reported in Supplemental Table 4.

Statistical testing was performed in SPSS version 26.0 (IBM Corp. Released 2019. IBM SPSS Statistics for Windows, version 26.0. Armonk, NY: IBM Corp) and in R (4.0.2).

Multiple imputation of missing values was performed using the SAS function PROC MI and Arbitrary Missing Patterns. Twenty imputed datasets were created to obtain smaller standard errors and to ensure that effect estimates were accurate. The results for each imputation were combined using SAS procedure PROC MIANALYZE. Level of significance was set to *p* < 0.05.

## Results

Patient baseline characteristics and baseline echocardiographic findings are presented in Table 1. The median (IQR) LVEF was 55% (47–60). The median (IQR) GLS was − 14.8% (− 17.8 to − 11.8%) with the IQR ranging from the lower limit of normal to reduced GLS.

**Table 1:** Baseline characteristics. *ACS*, acute coronary syndrome; *EDVi*, end diastolic volume indexed; *eGFR*, estimated glomerular filtration rate; *FPS*, frames per second; *GLS*, global longitudinal strain; *IQR*, interquartile range; *LVEF*, left ventricular ejection fraction; *NSTEMI*, non-ST-elevation myocardial infarction; *STEMI*, ST-elevation myocardial infarction; *data from RIKS-HIA *n* = 817

Baseline characteristics	
Age, years, median (IQR)	65 (58–72)
Male, *n* (%)	725 (77.0)
Active smoking*, *n* (%)	229 (28.0)
Diagnosis of ACS	
STEMI, *n* (%)	438 (46.7)
NSTEMI, *n* (%)	426 (45.4)
Unstable angina pectoris, *n* (%)	42 (4.5)
Unspecified ACS, *n* (%)	34 (3.6)
Revascularization*	
Angiography, *n* (%)	793 (97.4)
PCI, *n* (%)	673 (82.3)
CABG, *n* (%)	31 (3.8)
Treatment at discharge*	
Aspirin, *n* (%)	794 (97.2)
P2Y12 inhibitor, *n* (%)	735 (90.0)
Beta-blocker, *n* (%)	740 (90.6)
Statin, *n* (%)	789 (96.6)
Oral anticoagulant, *n* (%)	73 (9.0)
RAAS inhibitor, *n* (%)	683 (83.6)
Medical history	
Hypertension, *n* (%)	426 (45.3)
Diabetes mellitus, *n* (%)	144 (15.3)
Atrial fibrillation, *n* (%)	41 (4.4)
Heart failure, *n* (%)	35 (3.7)
History of stroke*, *n* (%)	38 (4.6)
History of myocardial infarction*, *n* (%)	153 (16.3)
Chronic kidney disease (eGFR < 60 ml/min/m^2^), *n* (%)	147 (15.6)
Echocardiographic parameters	
LVEF, median (IQR)	55 (47–60)
LV GLS, median (IQR)	− 14.8 (− 17.8 to − 11.8)
LV EDVi, ml/m^2^, median (IQR)	52.1 (44.9–61.7)
Frame rate, fps, median (IQR)	45.0 (36.0–51.2)

There were slight differences in baseline characteristics between included and excluded subjects (Supplemental Table 1). Excluded subjects had a higher rate of unspecified ACS and atrial fibrillation. There was also a trend towards a higher rate of stroke, heart failure diagnosis, and kidney disease among excluded subjects.

**Fig. 2:**
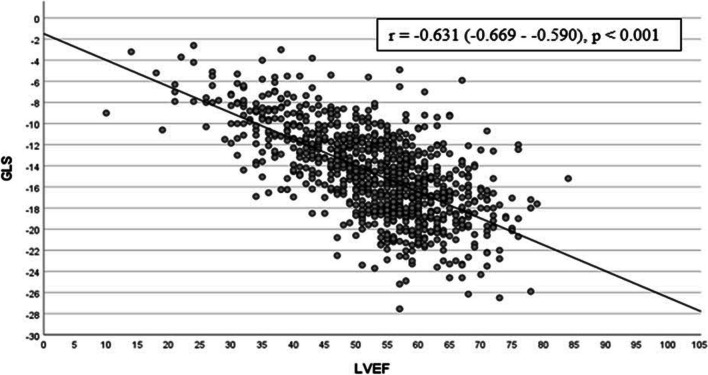
Correlation of LVEF and GLS determined by Spearman’s rho correlation coefficient

The negative correlation between LVEF and GLS is illustrated in Fig. 2 (*r* =  − 0.631, *p* < 0.001). During a median (IQR) follow-up time of 6.2 (4.6–8.0) years, the combined endpoint of HF re-admission and death was reached in 167 patients (17.7%) of which 114 (12.1%) patients died and 82 (8.7%) were re-admitted due to HF.

**Fig. 3:**
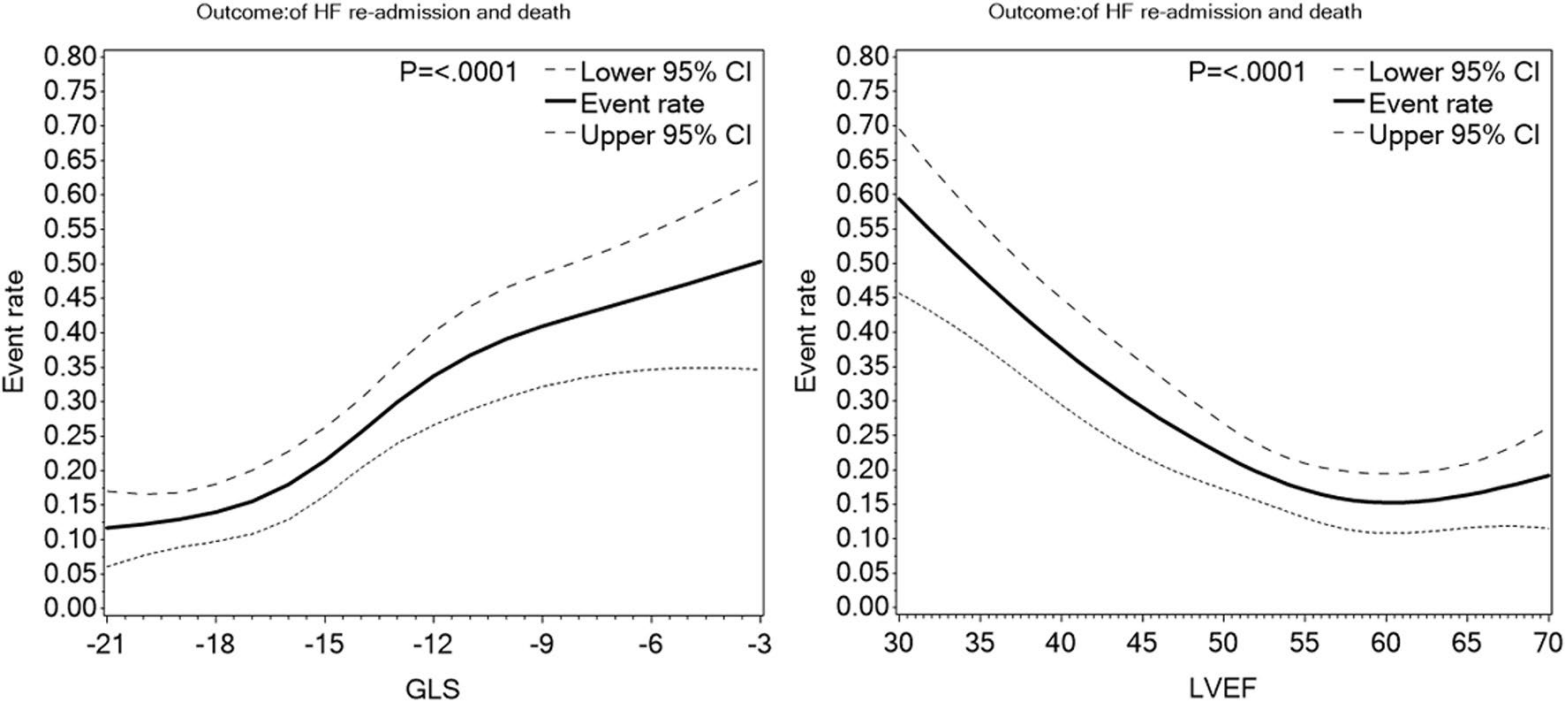
Spline plots of LVEF and GLS in relation to the combined outcome

As visualized in Fig. 3, LVEF appeared to have a J-shaped relation to the combined outcome with a flattening of the curve in patients with LVEF above 60%. GLS on the other hand appeared to present with more steadily increased risk with higher values.

In univariable analyses, GLS provided predictive information of both the combined endpoint and the individual endpoints (Table 2). GLS remained an independent predictor of the combined endpoint, with a hazard ratio (HR) of 1.068 (95% CI, 1.017–1.121) when adjusted for baseline characteristics and LVEF (Table 3). This was also found in prediction of HF re-admission. However, both GLS and LVEF lost their statistical significance as predictors of all-cause death in the multivariable model. The statistical significance in prediction of the combined outcome by GLS and LVEF appeared to be mainly driven by the prediction of HF re-hospitalization.

**Table 2:** Univariable Cox proportional hazards model for prediction of the combined and the single endpoints respectively. *CKD*, chronic kidney disease, defined as eGFR < 60 ml/min/m^2^; *CI*, confidence interval; *CHF*, congestive heart failure; *HR*, hazard ratio; *HT*, hypertension; *GLS*, global longitudinal strain; *LVEF*, left ventricular ejection fraction; *Prev. MI*, previous myocardial infarction; continuous variables (LVEF, GLS, age) have HR expressed in per unit change

	Combined endpoint			All-cause mortality			HF re-admission		
Variable	HR	95% CI	*p* value	HR	95% CI	*p* value	HR	95% CI	*p* value
LVEF	0.953	0.941–0.966	< 0.001	0.971	0.955–0.987	< 0.001	0.941	0.924–0.958	< 0.001
GLS	1.094	1.068–1.119	< 0.001	1.076	1.042–1.112	< 0.001	1.105	1.0071–1.141	< 0.001
Age	1.065	1.048–1.082	< 0.001	1.099	1.077–1.121	< 0.001	1.034	1.011–1.056	0.003
Male sex	0.969	0.694–1.421	0.969	0.842	0.555–1.278	0.420	1.629	0.901–2.947	0.107
Smoking	0.963	0.652–1.424	0.851	0.826	0.510–1.339	0.438	1.190	0.694–2.039	0.527
HT	1.722	1.263–2.349	< 0.001	2.103	1.431–3.090	< 0.001	1.610	1.036–2.504	0.034
Diabetes	1.852	1.335–2.570	< 0.001	1.862	1.254–2.764	0.002	1.682	1.051–2.693	0.030
CHF	4.672	3.066–7.120	< 0.001	5.236	3.224–8.503	< 0.001	3.413	1.849–6.900	< 0.001
Prev. MI	2.313	1.716–3.117	< 0.001	2.467	1.726–3.526	< 0.001	2.313	1.716–3.117	< 0.001
CKD	3.106	2.243–4.303	< 0.001	4.242	2.911–6.180	< 0.001	3.106	2.243–4.303	< 0.001

**Table 3:** Multivariable Cox proportional hazards model. *CKD*, chronic kidney disease, defined as eGFR < 60 ml/min/m^2^; *CI*, confidence interval; *CHF*, congestive heart failure; *HR*, hazard ratio; *HT*, hypertension; *GLS*, global longitudinal strain; *LVEF*, left ventricular ejection fraction; *Prev. MI*, previous myocardial infarction; continuous variables (LVEF, GLS, age) have HR expressed in per unit change

	Combined endpoint			All-cause mortality			HF re-admission		
Variable	HR	95% CI	*p* value	HR	95% CI	*p* value	HR	95% CI	*p* value
LVEF	0.980	0.962–0.998	0.031	0.985	0.964–1.008	0.194	0.971	0.948–0.996	0.022
GLS	1.068	1.017–1.121	0.009	1.029	0.970–1.091	0.343	1.075	1.003–1.153	0.042
Age	1.045	1.024–1.066	< 0.001	1.082	1.055–1.109	< 0.001	1.008	0.980–1.036	0.580
Male sex	0.978	0.667–1.434	0.910	1.018	0.648–1.599	0.939	0.707	0.377–1.329	0.282
HT	1.079	0.769–1.514	0.660	1.212	0.811–1.810	0.348	1.062	0.639–1.763	0.816
Diabetes	1.379	0.973–1.954	0.071	1.390	0.899–2.148	0.139	1.216	0.736–2.011	0.445
CHF	2.468	1.566–3.888	< 0.001	2.626	1.527–4.518	< 0.001	1.473	0.742–2.926	0.269
Prev. MI	1.370	0.945–1.985	0.096	1.259	0.823–1.957	0.281	1.858	1.134–3.043	0.014
CKD	1.381	0.935–2.040	0.105	1.535	0.981–2.402	0.061	1.492	0.836–2.664	0.176

**Fig. 4:**
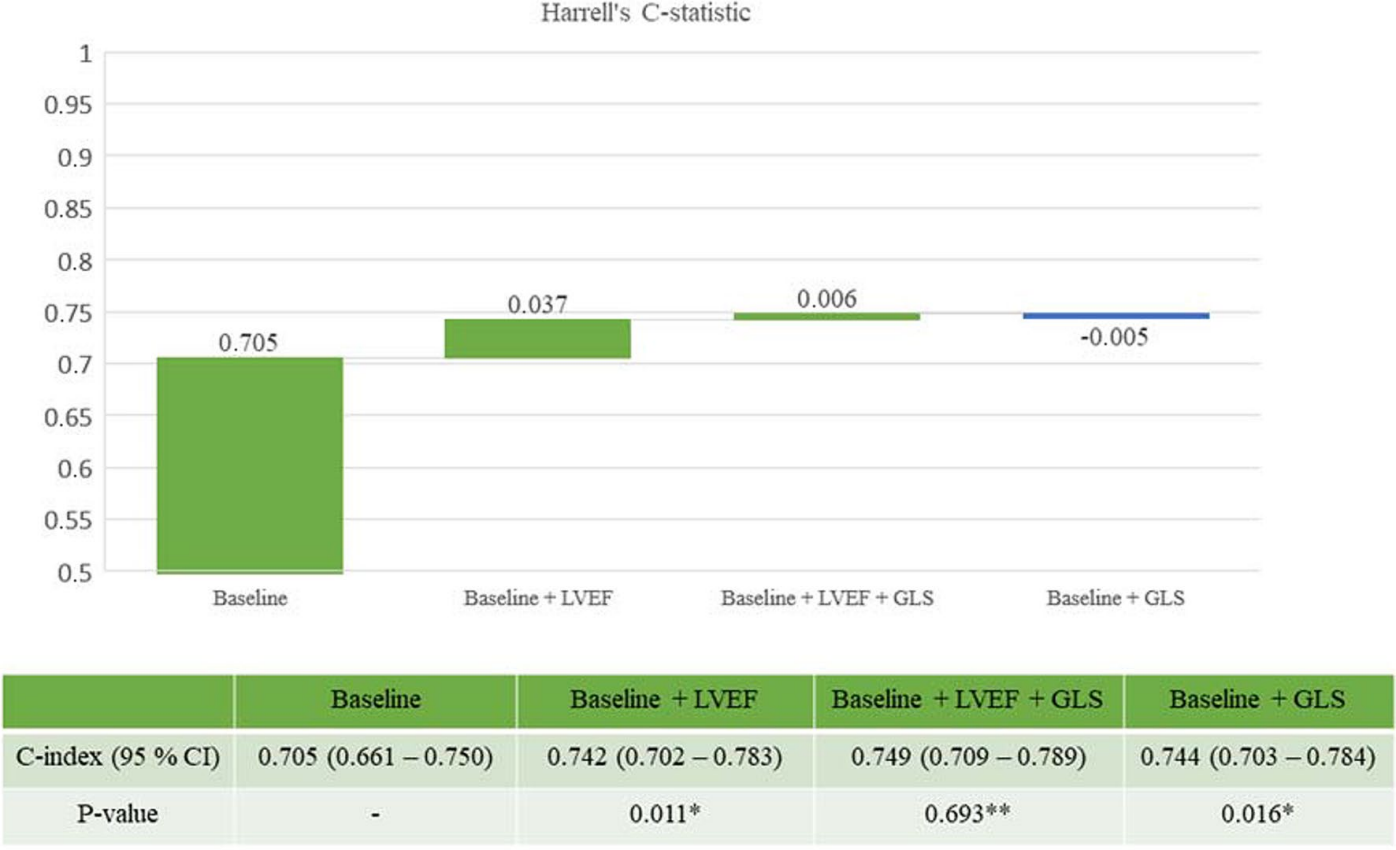
Model prediction of the combined endpoint. *Baseline is reference model; **baseline + LVEF is reference model. Baseline model: hypertension, previous MI, chronic kidney disease, heart failure, diabetes, sex, age

LVEF provided incremental prognostic information on top of clinical data with regard to the combined endpoint as measured by an improvement in Harrell’s C (Fig. 4). GLS also improved prognostication on top of the baseline model (Fig. 4). When GLS was added to a model of clinical data and LVEF, there was only a marginal increase in the C-statistic from 0.742 to 0.749 that did not meet statistical significance (*p* = 0.693). The subgroup analysis (Supplemental Table 2) demonstrated added prognostic value from imaging parameters (LVEF and GLS) particularly among STEMI patients.

**Fig. 5:**
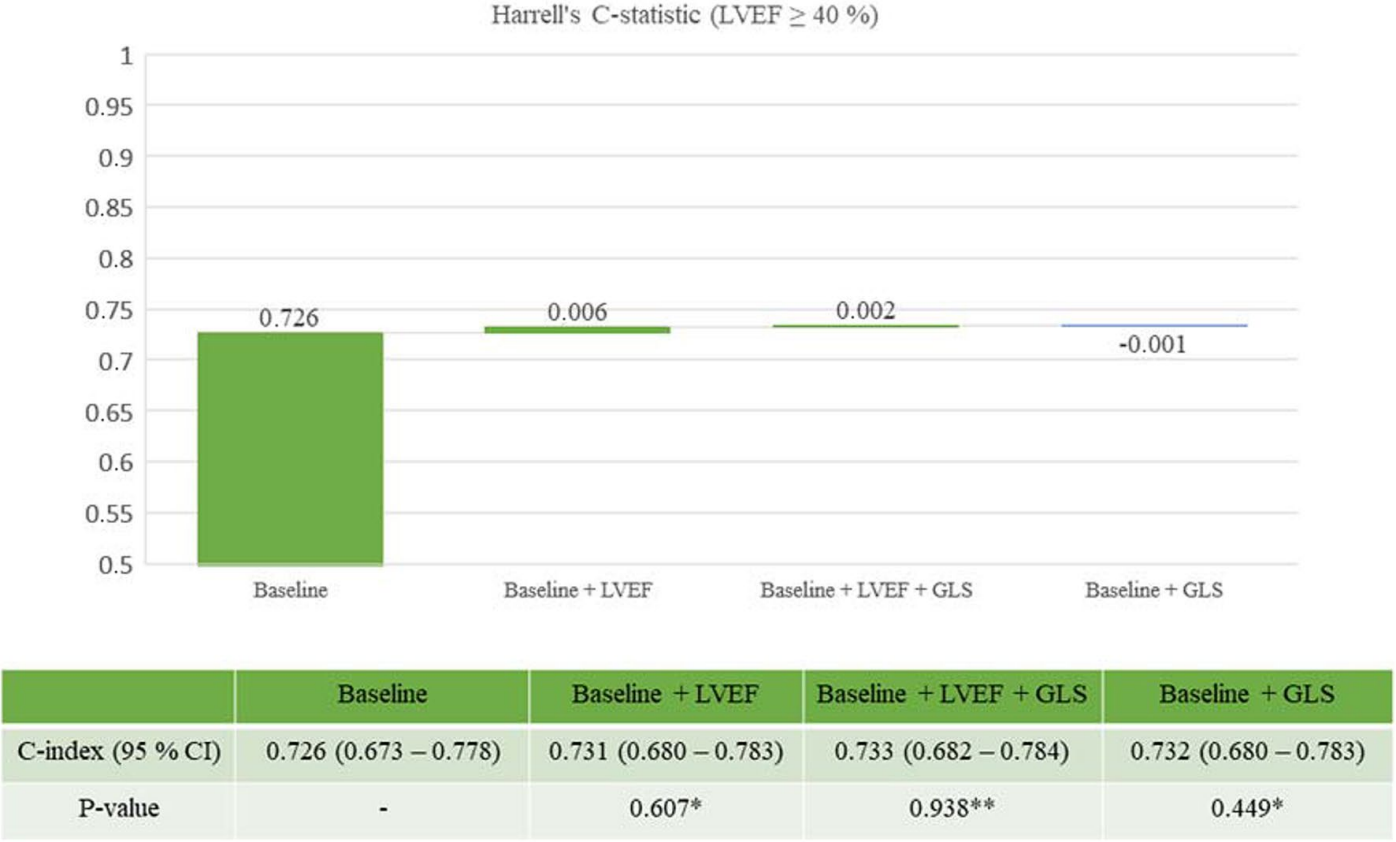
Model prediction of the combined endpoint in subjects with LVEF ≥ 40% (*n* = 820). Eighty-two (82) subjects reached the combined endpoint. *Baseline is reference model. **Baseline + LVEF is reference model. Baseline model: hypertension, previous MI, chronic kidney disease, heart failure, diabetes, sex, age

In the analysis stratified for subjects with LVEF > 40%, GLS provided a non-significant minimal increase in the C-statistic from 0.731 to 0.733 on top of LVEF (Fig. 5). Neither LVEF nor GLS improved the C-index with statistical significance on top of baseline data in patients with ejection fraction > 40%. Similar results were found when GLS was added to LVEF and the second baseline model presented in Supplemental Table 3.

**Fig. 6:**
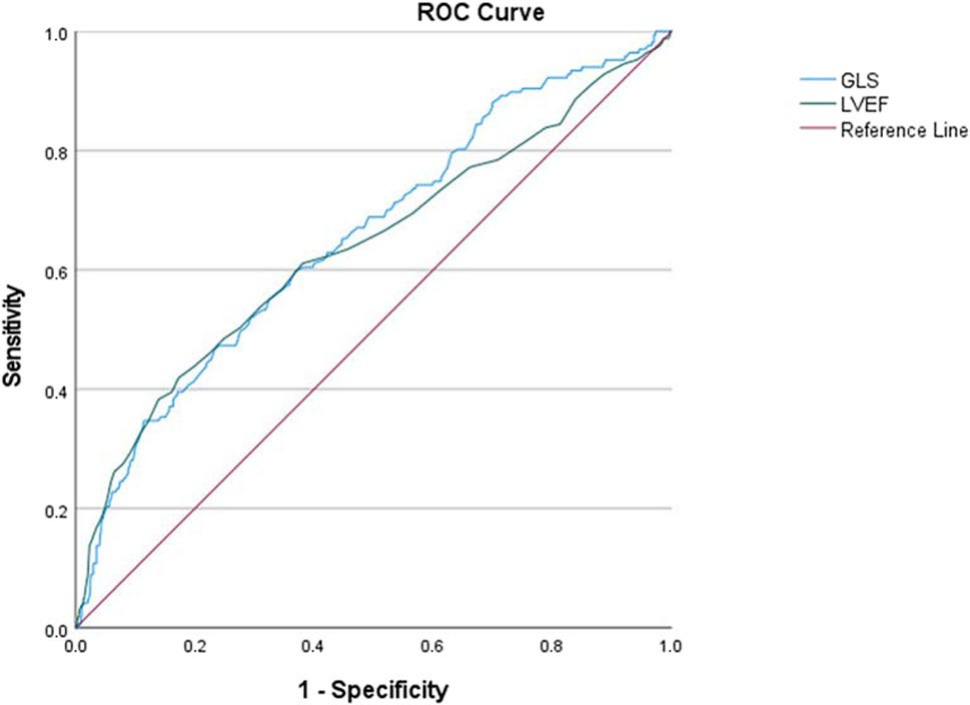
Receiver operating characteristic (ROC) curve of GLS and LVEF as predictors of the combined outcome. Area under curve (AUC) for GLS is 0.656 (95% CI (0.609–0.703)) and for LVEF 0.638 (95% CI (0.587–0.689))

From the unadjusted ROC analysis in Fig. 6, the optimal predictive cut-off value of GLS was − 13.8% with a sensitivity of 60% and specificity of 63%. GLS >  − 13.8% had an unadjusted HR of 2.658 (95% CI 1.947–3.629) for the combined endpoint. The corresponding cut-off for LVEF was 53% with a sensitivity of 61% and specificity of 62%.

## Discussion

Global longitudinal strain was an independent long-term predictor of the composite endpoint all-cause death and heart failure re-hospitalization after ACS. An unadjusted GLS more than − 13.8% was associated with a 73% probability of meeting the combined outcome before someone with a lower GLS [12]. However, the step-wise addition of GLS to the prognostic model of clinical data and LVEF resulted in a non-significant marginal change in the C-index.

Previous studies have reported independent prognostic value of GLS in short- to long-term follow-up settings after ACS [13–16]. The results from our study strengthen the support for GLS as a long-term prognostic marker in ACS patients although GLS provides no meaningful information when LVEF is known.

LVEF is recognized as a poor predictor of outcome in subjects with preserved or mildly reduced ejection fraction (LVEF > 40%) [17, 18]. In patients with preserved LVEF and non-ischemic heart diseases, such as hypertrophic cardiomyopathy or severe aortic stenosis, previous studies have supported GLS as a superior and more sensitive marker of adverse outcome [19, 20]. Our finding, with a non-significant minimal improvement of the C-index from the addition of GLS to the predictive model in subjects with LVEF > 40% (Fig. 5), suggests that GLS does not provide the same improved long-term prognostic information in ACS as have been reported in non-ischemic cardiac disease.

As presented in Supplemental Table 2, only GLS emerged with statistical significance in prediction of the combined endpoint among men and only LVEF emerged with statistical significance among women. A closer look at sex stratified baseline data (Supplemental Table 4) revealed that women had significantly higher LVEF than men (median (IQR): 57.0 (49.0 to 63.8) and 54.0 (46.0 to 59.5), respectively). This may be a clue to the sex difference in LVEF and GLS performance—since findings in the LVEF stratified analyses indicated greater prognostic value of GLS in subjects with lower LVEF.

Previous investigations in post-MI subjects have indicated a prognostic value of GLS with reference to statistical significance; however, they rarely take clinical utility into account [3, 14, 15]. The large prospective study on subjects with LVEF > 40% after STEMI by Ersbøll et al. demonstrated independent prognostic properties of GLS in a midterm follow-up setting (median 30 months, IQR 24.3 to 32.8) although they did not include LVEF in the multivariable analysis with clinical data, hindering a comparison of incremental prognostic value [16].

The optimal GLS cut-off, − 13.8%, from the ROC analysis was slightly lower than cut-off values previously reported. One study with post-MI subjects from South Korea with a mean follow-up time of 38.6 ± 19.2 months presented an optimal cut-off of − 9.9% [15]. Another study on GLS as predictor of 30-day outcome post-STEMI in subjects with preserved EF reported an optimal cut-off of − 12.7 and a study of 6-month outcome in unselected STEMI patients reported an optimal cut-off − 12.5% [13, 14]. These differences in cut-off values may in part be explained by inter-software variations. However, TomTec has been reported to render both lower and higher GLS values compared to other software in separate studies, preventing any definite conclusion of this impact [21, 22]. The shorter follow-up time of the previous studies may contribute to a higher GLS cut-off given that the Cox regression analysis suggests a relationship between GLS and time to adverse outcome.

Median GLS was reduced below normal in this study sample (− 14.8%; − 17.8 to − 11.8) yet median LVEF remained preserved [23, 24]. This may be explained by the notion that early myocardial injury predominantly affects the longitudinal subendocardial fibers contributing to GLS with sparing of the midwall circumferential fibers that mainly contribute to LVEF [25, 26]. GLS from the TomTec software has been reported to have a lower limit of normal at − 18% [22]. It remains unexpected that even though GLS on average was reduced, it did not provide a greater improvement in prognostication when added to the model with clinical data and LVEF in any of the analyzed populations (Figs. 4 and 5). This could arguably be explained by temporary myocardial stunning that restitutes over time. Since there are no follow-up examinations in this material, the impact of stunning in the sub-acute setting remains uncertain.

Previous studies have suggested that GLS could be a more robust and reproducible measurement than LVEF although conflicting findings have been reported [27, 28]. Indeed, should GLS consistently prove to be the more robust metric this would strengthen the argument for GLS as the preferred echo measurement in ACS risk assessments. However, randomized prospective intervention studies guided by GLS are still needed for its adoption in clinical decision-making.

### Limitations

There are several limitations in the current study that need to be addressed. First, the included patients were recruited between 2008 and 2014, and this was before a widespread adoption of GLS assessments in the clinical setting. Consequently, imaging protocols were not adapted for speckle tracking analysis and several subjects lacked an apical three-chamber view which only allowed for biplane assessments of GLS. Furthermore, there was a substantial number of excluded subjects due to insufficient image quality and loss of raw data.

The number of excluded patients due to insufficient image quality was higher than expected and this cannot easily be explained. It has previously been reported that roughly 12% of echocardiographic examinations are unsuitable for quantitative LVEF assessment without injected contrast and there were no contrast examinations included in this material [29]. Why the echocardiographic raw data of 234 subjects was lost along the way can only be speculated upon; however, it can be summarized as a weakness attributable to the retrospective design.

Second, the retrospective design has also limited clinical data acquisition, which prevented adjustments for confounding parameters such as blood pressure at examination and location of myocardial insult. High sensitivity troponins carry important prognostic information in ACS; however, inter-center assay differences prevented the inclusion of troponin as a continuous variable in the analysis. Troponins should preferably be included in future prognostic models when available [30].

Third, LVEF and GLS are both assessments of global myocardial function and any regional injury without global implications is therefore not accounted for. The speckle tracking technique allows regional functional assessment by calculation of mechanical dispersion between myocardial segments. Such assessments could possibly further strengthen echocardiographic prognostication post-MI [31, 32]. Evaluation of mechanical dispersion was, however, beyond the scope of this analysis and its clinical application as a predictor may be limited [33].

Lastly, myocardial stunning may be a confounding factor in the assessment of systolic function the first days following ACS. A repeated echocardiographic examination a few weeks after admission would more accurately correspond to irreversible injury. There is however no routine to repeat examinations among subjects with LVEF > 35% in Sweden. Consequently, that analysis is not possible in the current cohort.

## Conclusion

In this large real-world cohort of ACS patients, recruited between 2008 and 2014 with predominantly normal or near-normal ejection fraction, both LVEF and GLS emerged as independent long-term risk predictors of combined all-cause mortality and heart failure re-hospitalizations. However, there was no significant improvement in outcome prediction from GLS when LVEF was already known.

## Supplementary Information

Below is the link to the electronic supplementary material.

Supplementary file1 (DOCX 58.4 KB)

## Data Availability

The dataset analyzed in this study is not publicly available due to Swedish patient privacy and secrecy laws regulating access to SWEDEHEART. Researchers are able to access the data at Uppsala Clinical Research Center upon reasonable request and under the provision that the data is accessed onsite and does not leave Uppsala University. This request can be sent to info@ucr.uu.se.
