# Supplementary material for: Global longitudinal strain in long-term risk prediction after acute coronary syndrome: an investigation of added prognostic value to ejection fraction
Source: Clin Res Cardiol. 2024 Mar 25;114(6):709–18. doi: 10.1007/s00392-024-02439-w (PMC12089238; doi:10.1007/s00392-024-02439-w)
Supplement: Supplementary file 1 — Supplementary file1 (DOCX 58.4 KB) [file 392_2024_2439_MOESM1_ESM.docx]

Supplemental material:

Global longitudinal strain in long-term risk prediction after acute coronary syndrome: An investigation of added prognostic value to ejection fraction

Journal: Clinical Research in Cardiology

Joel Lenell^a,b^, Bertil Lindahl^a,b^, David Erlinge^c^, Tomas Jernberg^d^, Jonas Spaak^d^, Tomasz Baron^a,b^

^a^Department of Medical Sciences, Cardiology, Uppsala University, Uppsala, Sweden; ^b^Uppsala Clinical Research Center, Uppsala University, Uppsala, Sweden; ^c^Dept. of Clinical Sciences, Cardiology, Lund University, Skåne University Hospital, Lund, Sweden; ^d^Dept. of Clinical Sciences, Division of Cardiovascular Medicine, Danderyd Hospital, Karolinska Institutet, Stockholm, Sweden

Corresponding author:

Joel Lenell

Email: [joel.lenell@medsci.uu.se](mailto:joel.lenell@medsci.uu.se)

| Baseline characteristics | Included subjects | Excluded subjects | | p-value |
| --- | --- | --- | --- | --- |
| Age. years. median (IQR) | 65 (58-72) | 66 (57-74) | 0.662 | |
| Male. n (%) | 725 (77.0) | 324 (74.3) | 0.236 | |
| Active smoking* n (%) | 229 (28.0) | 99 (22.7) | 0.792 | |
| Diagnosis of ACS. n (%) |  |  |  | |
| STEMI | 438 (46.7) | 193 (44.3) | 0.492 | |
| NSTEMI | 426 (45.4) | 184 (42.2) | 0.347 | |
| Unstable angina pectoris | 42 (4.5) | 23 (5.3) | 0.127 | |
| Unspecified ACS | 34 (3.6) | 43 (9.7) | 0.030 | |
| Revascularization. n (%)* |  |  |  | |
| Angiography | 793 (97.4) | 377 (92.6) | 0.017 | |
| PCI | 673 (82.3) | 320 (78.6) | 0.245 | |
| CABG | 31 (3.8) | 29 (7.1) | 0.008 | |
| Treatment at discharge. n (%)* |  |  |  | |
| Aspirin | 794 (97.2) | 376 (95.2) | 0.134 | |
| P2Y12-inhibitor | 735 (90.0) | 342 (86.6) | 0.210 | |
| Betablocker | 740 (90.6) | 354 (89.6) | 0.328 | |
| Statin | 789 (96.6) | 371 (93.9) | 0.102 | |
| Oral anticoagulant | 73 (9.0) | 42 (10.6) | 0.334 | |
| RAAS-inhibitor | 683 (83.6) | 325 (82.3) | 0.744 | |
| Medical history. n (%) |  |  |  | |
| Hypertension | 426 (45.3) | 204 (46.8) | 0.464 | |
| Diabetes mellitus | 144 (15.3) | 101 (23.2) | 0.439 | |
| Atrial fibrillation | 41 (4.4) | 56 (12.8) | 0.040 | |
| Heart failure | 35 (3.7) | 35 (8.0) | 0.064 | |
| History of stroke | 38 (4.6) | 40 (9.2) | 0.062 | |
| History of myocardial infarction | 153 (16.3) | 76 (19.1) | 0.932 | |
| Chronic kidney disease (GFR <60ml/min) | 147 (15.6) | 86 (19.7) | 0.057 | |

Supplemental table 1.

Title: Baseline characteristics among patients with, and those excluded without, readable echocardiographic images

Supplemental table 1. Comparison of baseline characteristics between patients with and without available echo data. Non-parametric scale variables were analyzed with Mann Whitney U test and categorical variables were analyzed with Chi^2^ statistics. * data from RIKS-HIA, N in inclusion group = 817.

Supplemental table 2.

Title: Subgroup analyses stratified for infarction type, sex, age, and category of LVEF

Supplemental table 2. Stepwise multivariable Cox proportional hazard models by stratification. Endpoint is combined all-cause death and heart failure re-hospitalization.

Chronic kidney disease (CKD); C-statistic (C-stat); Global longitudinal strain (GLS); Heart failure (HF); Left ventricular ejection fraction (LVEF); Previous myocardial infarction (Previous MI)

| Subgroup: STEMI |  |  |  |  |
| --- | --- | --- | --- | --- |
|  |  |  |  |  |
| Variable | n | HR (95% CI) | p-value | c-stat (95% CI) |
|  | | | | |
| Age | 438 | 1.040 (1.009-1.072) | 0.012 | 0.630 (0.557-0.703) |
| Previous MI |  | 1.330 (0.732-2.417) | 0.349 |  |
| CKD |  | 1.343 (0.725-2.487) | 0.349 |  |
| Diabetes |  | 1.575 (0.905-2.739) | 0.108 |  |
| Heart failure |  | 2.653 (1.116-6.306) | 0.027 |  |
| Hypertension |  | 0.911 (0.548-1.516) | 0.721 |  |
| Male sex |  | 1.057 (0.623-1.794) | 0.837 |  |
|  | | | | |
| LVEF | 438 | 0.930 (0.909-0.950) | <.001 | 0.754 (0.692-0.816) |
| Age |  | 1.041 (1.009-1.073) | 0.011 |  |
| Previous MI |  | 1.268 (0.677-2.373) | 0.459 |  |
| CKD |  | 1.451 (0.798-2.639) | 0.222 |  |
| Diabetes |  | 1.383 (0.805-2.375) | 0.240 |  |
| Heart failure |  | 2.808 (1.316-5.991) | 0.008 |  |
| Hypertension |  | 1.036 (0.630-1.703) | 0.889 |  |
| Male sex |  | 1.050 (0.613-1.799) | 0.859 |  |
|  | | | | |
| GLS | 438 | 1.207 (1.121-1.299) | <.001 | 0.747 (0.687-0.808) |
| Age |  | 1.029 (0.999-1.060) | 0.062 |  |
| Previous MI |  | 1.487 (0.823-2.688) | 0.189 |  |
| CKD |  | 1.230 (0.677-2.234) | 0.497 |  |
| Diabetes |  | 1.210 (0.691-2.119) | 0.505 |  |
| Heart failure |  | 4.315 (2.046-9.099) | 0.000 |  |
| Hypertension |  | 1.058 (0.635-1.765) | 0.828 |  |
| Male sex |  | 1.102 (0.633-1.918) | 0.731 |  |
|  | | | | |
| LVEF | 438 | 0.945 (0.920-0.971) | <.001 | 0.760 (0.697-0.823) |
| GLS |  | 1.077 (0.992-1.169) | 0.078 |  |
| Age |  | 1.037 (1.006-1.070) | 0.020 |  |
| Previous MI |  | 1.312 (0.694-2.480) | 0.403 |  |
| CKD |  | 1.342 (0.733-2.457) | 0.341 |  |
| Diabetes |  | 1.298 (0.754-2.236) | 0.347 |  |
| Heart failure |  | 3.439 (1.595-7.417) | 0.002 |  |
| Hypertension |  | 1.077 (0.656-1.768) | 0.770 |  |
| Male sex |  | 1.037 (0.596-1.805) | 0.897 |  |
|  |  |  |  |  |
|  |  |  |  |  |
| Subgroup: NSTEMI |  |  |  |  |
|  |  |  |  |  |
| Variable | n | HR (95% CI) | p-value | c-stat (95% CI) |
|  |  |  |  |  |
| Age | 426 | 1.042 (1.012-1.074) | 0.006 | 0.747 (0.686-0.808) |
| Previous MI |  | 1.401 (0.867-2.263) | 0.169 |  |
| CKD |  | 2.107 (1.198-3.706) | 0.010 |  |
| Diabetes |  | 1.568 (0.921-2.670) | 0.098 |  |
| Heart failure |  | 2.694 (1.445-5.024) | 0.002 |  |
| Hypertension |  | 0.983 (0.587-1.645) | 0.947 |  |
| Male sex |  | 0.890 (0.500-1.585) | 0.692 |  |
|  | | | | |
| LVEF | 426 | 0.982 (0.963-1.002) | 0.081 | 0.755 (0.694-0.815) |
| Age |  | 1.045 (1.014-1.076) | 0.004 |  |
| Previous MI |  | 1.378 (0.832-2.283) | 0.213 |  |
| CKD |  | 1.976 (1.120-3.484) | 0.019 |  |
| Diabetes |  | 1.587 (0.933-2.698) | 0.088 |  |
| Heart failure |  | 2.451 (1.317-4.564) | 0.005 |  |
| Hypertension |  | 0.996 (0.586-1.693) | 0.987 |  |
| Male sex |  | 0.975 (0.548-1.737) | 0.932 |  |
|  | | | | |
| GLS | 426 | 1.067 (1.009-1.129) | 0.024 | 0.753 (0.691-0.816) |
| Age |  | 1.038 (1.008-1.069) | 0.014 |  |
| Previous MI |  | 1.376 (0.836-2.265) | 0.209 |  |
| CKD |  | 1.996 (1.144-3.484) | 0.015 |  |
| Diabetes |  | 1.533 (0.912-2.575) | 0.107 |  |
| Heart failure |  | 2.551 (1.419-4.586) | 0.002 |  |
| Hypertension |  | 1.013 (0.590-1.740) | 0.962 |  |
| Male sex |  | 0.923 (0.517-1.650) | 0.788 |  |
|  | | | | |
| LVEF | 426 | 0.995 (0.970-1.021) | 0.699 | 0.754 (0.692-0.816) |
| GLS |  | 1.057 (0.983-1.136) | 0.134 |  |
| Age |  | 1.039 (1.009-1.070) | 0.012 |  |
| Previous MI |  | 1.376 (0.832-2.275) | 0.213 |  |
| CKD |  | 1.970 (1.123-3.454) | 0.018 |  |
| Diabetes |  | 1.542 (0.916-2.598) | 0.103 |  |
| Heart failure |  | 2.504 (1.379-4.545) | 0.003 |  |
| Hypertension |  | 1.015 (0.590-1.745) | 0.957 |  |
| Male sex |  | 0.944 (0.521-1.711) | 0.851 |  |
|  |  |  |  |  |
|  |  |  |  |  |
| Subgroup: Men |  |  |  |  |
|  |  |  |  |  |
| Variable | n | HR (95% CI) | p-value | c-stat (95% CI) |
|  |  |  |  |  |
| Age | 725 | 1.059 (1.035-1.083) | <.001 | 0.711 (0.662-0.761) |
| Previous MI |  | 1.508 (1.052-2.162) | 0.026 |  |
| CKD |  | 1.333 (0.871-2.039) | 0.185 |  |
| Diabetes |  | 1.961 (1.341-2.869) | 0.001 |  |
| Heart failure |  | 2.085 (1.220-3.562) | 0.007 |  |
| Hypertension |  | 0.998 (0.687-1.450) | 0.991 |  |
|  | | | | |
| LVEF | 725 | 0.968 (0.952-0.984) | <.001 | 0.751 (0.707-0.795) |
| Age |  | 1.060 (1.036-1.084) | <.001 |  |
| Previous MI |  | 1.416 (0.948-2.115) | 0.090 |  |
| CKD |  | 1.242 (0.799-1.932) | 0.336 |  |
| Diabetes |  | 1.898 (1.288-2.796) | 0.001 |  |
| Heart failure |  | 1.828 (1.027-3.255) | 0.041 |  |
| Hypertension |  | 1.043 (0.709-1.537) | 0.829 |  |
|  | | | | |
| GLS | 725 | 1.128 (1.076-1.182) | <.001 | 0.761 (0.719-0.803) |
| Age |  | 1.049 (1.026-1.074) | <.001 |  |
| Previous MI |  | 1.469 (0.991-2.177) | 0.055 |  |
| CKD |  | 1.263 (0.824-1.935) | 0.283 |  |
| Diabetes |  | 1.743 (1.190-2.553) | 0.004 |  |
| Heart failure |  | 2.165 (1.319-3.555) | 0.002 |  |
| Hypertension |  | 1.036 (0.702-1.530) | 0.858 |  |
|  | | | | |
| LVEF | 725 | 0.989 (0.970-1.009) | 0.291 | 0.764 (0.722-0.806) |
| GLS |  | 1.103 (1.040-1.169) | 0.001 |  |
| Age |  | 1.051 (1.027-1.076) | <.001 |  |
| Previous MI |  | 1.445 (0.966-2.161) | 0.073 |  |
| CKD |  | 1.236 (0.802-1.906) | 0.337 |  |
| Diabetes |  | 1.765 (1.203-2.590) | 0.004 |  |
| Heart failure |  | 2.064 (1.226-3.474) | 0.006 |  |
| Hypertension |  | 1.049 (0.709-1.553) | 0.811 |  |
|  |  |  |  |  |
|  |  |  |  |  |
| Subgroup: Women |  |  |  |  |
|  |  |  |  |  |
| Variable | n | HR (95% CI) | p-value | c-stat (95% CI) |
|  | | | | |
| Age | 216 | 1.024 (0.984-1.066) | 0.239 | 0.729 (0.633-0.826) |
| Previous MI |  | 1.656 (0.708-3.871) | 0.245 |  |
| CKD |  | 2.414 (1.080-5.398) | 0.032 |  |
| Diabetes |  | 0.538 (0.224-1.294) | 0.166 |  |
| Heart failure |  | 3.738 (1.644-8.499) | 0.002 |  |
| Hypertension |  | 1.103 (0.558-2.176) | 0.779 |  |
|  | | | | |
| LVEF | 216 | 0.955 (0.920-0.991) | 0.015 | 0.739 (0.646-0.831) |
| Age |  | 1.031 (0.991-1.073) | 0.128 |  |
| Previous MI |  | 1.891 (0.781-4.577) | 0.158 |  |
| CKD |  | 2.045 (0.928-4.507) | 0.076 |  |
| Diabetes |  | 0.490 (0.195-1.232) | 0.130 |  |
| Heart failure |  | 3.165 (1.390-7.207) | 0.006 |  |
| Hypertension |  | 1.306 (0.659-2.589) | 0.444 |  |
|  | | | | |
| GLS | 216 | 1.048 (0.969-1.133) | 0.238 | 0.736 (0.641-0.831) |
| Age |  | 1.018 (0.978-1.060) | 0.374 |  |
| Previous MI |  | 1.557 (0.640-3.788) | 0.329 |  |
| CKD |  | 2.271 (1.007-5.126) | 0.048 |  |
| Diabetes |  | 0.531 (0.226-1.250) | 0.148 |  |
| Heart failure |  | 3.791 (1.674-8.584) | 0.001 |  |
| Hypertension |  | 1.179 (0.584-2.379) | 0.647 |  |
|  | | | | |
| LVEF | 216 | 0.945 (0.900-0.992) | 0.023 | 0.742 (0.650-0.833) |
| GLS |  | 0.959 (0.866-1.062) | 0.423 |  |
| Age |  | 1.037 (0.994-1.082) | 0.093 |  |
| Previous MI |  | 2.022 (0.790-5.175) | 0.142 |  |
| CKD |  | 2.149 (0.968-4.767) | 0.060 |  |
| Diabetes |  | 0.485 (0.186-1.264) | 0.139 |  |
| Heart failure |  | 2.973 (1.234-7.164) | 0.015 |  |
| Hypertension |  | 1.261 (0.623-2.550) | 0.519 |  |
|  |  |  |  |  |
|  |  |  |  |  |
| Subgroup: Age < 65 |  |  |  |  |
|  |  |  |  |  |
| Variable | n | HR (95% CI) | p-value | c-stat (95% CI) |
|  | | | | |
| Age | 434 | 1.025 (0.970-1.084) | 0.375 | 0.668 (0.584-0.751) |
| Previous MI |  | 1.300 (0.624-2.712) | 0.484 |  |
| CKD |  | 2.149 (0.669-6.903) | 0.199 |  |
| Diabetes |  | 2.695 (1.431-5.075) | 0.002 |  |
| Heart failure |  | 3.466 (1.097-10.96) | 0.034 |  |
| Hypertension |  | 0.719 (0.377-1.372) | 0.317 |  |
| Male sex |  | 0.875 (0.399-1.916) | 0.738 |  |
|  | | | | |
| LVEF | 434 | 0.935 (0.905-0.966) | <.001 | 0.743 (0.662-0.825) |
| Age |  | 1.023 (0.971-1.078) | 0.387 |  |
| Previous MI |  | 1.151 (0.552-2.401) | 0.707 |  |
| CKD |  | 1.629 (0.571-4.646) | 0.362 |  |
| Diabetes |  | 2.626 (1.456-4.735) | 0.001 |  |
| Heart failure |  | 2.189 (0.767-6.250) | 0.143 |  |
| Hypertension |  | 0.769 (0.405-1.460) | 0.422 |  |
| Male sex |  | 1.131 (0.540-2.373) | 0.744 |  |
|  | | | | |
| GLS | 434 | 1.156 (1.068-1.250) | <.001 | 0.714 (0.627-0.801) |
| Age |  | 1.028 (0.973-1.087) | 0.319 |  |
| Previous MI |  | 1.327 (0.638-2.760) | 0.450 |  |
| CKD |  | 1.971 (0.698-5.560) | 0.200 |  |
| Diabetes |  | 2.246 (1.216-4.149) | 0.010 |  |
| Heart failure |  | 3.371 (1.331-8.542) | 0.010 |  |
| Hypertension |  | 0.682 (0.357-1.304) | 0.247 |  |
| Male sex |  | 1.032 (0.485-2.196) | 0.934 |  |
|  | | | | |
| LVEF | 434 | 0.950 (0.907-0.995) | 0.028 | 0.736 (0.652-0.819) |
| GLS |  | 1.056 (0.951-1.173) | 0.308 |  |
| Age |  | 1.025 (0.972-1.080) | 0.360 |  |
| Previous MI |  | 1.186 (0.566-2.482) | 0.651 |  |
| CKD |  | 1.642 (0.583-4.623) | 0.348 |  |
| Diabetes |  | 2.482 (1.375-4.480) | 0.003 |  |
| Heart failure |  | 2.417 (0.849-6.881) | 0.098 |  |
| Hypertension |  | 0.748 (0.389-1.440) | 0.385 |  |
| Male sex |  | 1.144 (0.546-2.395) | 0.722 |  |
|  |  |  |  |  |
|  |  |  |  |  |
| Subgroup: Age ≥ 65 |  |  |  |  |
|  |  |  |  |  |
| Variable | n | HR (95% CI) | p-value | c-stat (95% CI) |
|  | | | | |
| Age | 507 | 1.070 (1.039-1.103) | <.001 | 0.697 (0.648-0.746) |
| Previous MI |  | 1.452 (0.992-2.127) | 0.055 |  |
| CKD |  | 1.371 (0.905-2.076) | 0.137 |  |
| Diabetes |  | 1.238 (0.810-1.891) | 0.324 |  |
| Heart failure |  | 2.535 (1.533-4.193) | 0.000 |  |
| Hypertension |  | 1.164 (0.783-1.732) | 0.453 |  |
| Male sex |  | 0.786 (0.508-1.216) | 0.279 |  |
|  | | | | |
| LVEF | 507 | 0.972 (0.955-0.988) | 0.001 | 0.727 (0.680-0.774) |
| Age |  | 1.076 (1.044-1.108) | <.001 |  |
| Previous MI |  | 1.388 (0.915-2.106) | 0.123 |  |
| CKD |  | 1.266 (0.826-1.940) | 0.279 |  |
| Diabetes |  | 1.204 (0.788-1.839) | 0.391 |  |
| Heart failure |  | 2.426 (1.456-4.042) | 0.001 |  |
| Hypertension |  | 1.256 (0.831-1.897) | 0.280 |  |
| Male sex |  | 0.890 (0.571-1.387) | 0.607 |  |
|  | | | | |
| GLS | 507 | 1.096 (1.046-1.149) | 0.000 | 0.728 (0.682-0.774) |
| Age |  | 1.066 (1.035-1.099) | <.001 |  |
| Previous MI |  | 1.364 (0.908-2.049) | 0.135 |  |
| CKD |  | 1.282 (0.841-1.952) | 0.248 |  |
| Diabetes |  | 1.150 (0.759-1.742) | 0.509 |  |
| Heart failure |  | 2.660 (1.648-4.293) | <.0001 |  |
| Hypertension |  | 1.282 (0.843-1.950) | 0.246 |  |
| Male sex |  | 0.803 (0.514-1.254) | 0.335 |  |
|  | | | | |
| LVEF | 507 | 0.986 (0.965-1.006) | 0.174 | 0.732 (0.686-0.778) |
| GLS |  | 1.065 (1.007-1.127) | 0.028 |  |
| Age |  | 1.070 (1.038-1.103) | <.001 |  |
| Previous MI |  | 1.361 (0.895-2.068) | 0.149 |  |
| CKD |  | 1.250 (0.816-1.913) | 0.305 |  |
| Diabetes |  | 1.159 (0.763-1.761) | 0.489 |  |
| Heart failure |  | 2.567 (1.572-4.193) | 0.000 |  |
| Hypertension |  | 1.298 (0.851-1.978) | 0.226 |  |
| Male sex |  | 0.856 (0.543-1.350) | 0.504 |  |
|  |  |  |  |  |
|  |  |  |  |  |
| Subgroup: LVEF ≥ 40 |  |  |  |  |
|  |  |  |  |  |
| Variable | n | HR (95% CI) | p-value | c-stat (95% CI) |
|  | | | | |
| Age | 820 | 1.055 (1.032-1.079) | <.001 | 0.726 (0.673-0.778) |
| Previous MI |  | 1.741 (1.206-2.514) | 0.003 |  |
| CKD |  | 1.717 (1.098-2.687) | 0.018 |  |
| Diabetes |  | 1.372 (0.905-2.080) | 0.136 |  |
| Heart failure |  | 2.615 (1.525-4.483) | 0.001 |  |
| Hypertension |  | 0.964 (0.663-1.404) | 0.850 |  |
| Male sex |  | 0.847 (0.544-1.320) | 0.464 |  |
|  | | | | |
| LVEF | 820 | 0.974 (0.947-1.001) | 0.060 | 0.731 (0.680-0.783) |
| Age |  | 1.057 (1.034-1.081) | <.001 |  |
| Previous MI |  | 1.814 (1.251-2.632) | 0.002 |  |
| CKD |  | 1.672 (1.067-2.620) | 0.025 |  |
| Diabetes |  | 1.319 (0.870-1.998) | 0.192 |  |
| Heart failure |  | 2.568 (1.526-4.323) | 0.000 |  |
| Hypertension |  | 0.992 (0.676-1.454) | 0.966 |  |
| Male sex |  | 0.877 (0.560-1.372) | 0.565 |  |
|  | | | | |
| GLS | 820 | 1.051 (1.003-1.102) | 0.036 | 0.732 (0.680-0.783) |
| Age |  | 1.051 (1.028-1.075) | <.001 |  |
| Previous MI |  | 1.756 (1.215-2.537) | 0.003 |  |
| CKD |  | 1.714 (1.100-2.671) | 0.017 |  |
| Diabetes |  | 1.310 (0.869-1.975) | 0.197 |  |
| Heart failure |  | 2.630 (1.579-4.382) | 0.000 |  |
| Hypertension |  | 0.978 (0.667-1.433) | 0.909 |  |
| Male sex |  | 0.839 (0.534-1.319) | 0.447 |  |
|  | | | | |
| LVEF | 820 | 0.982 (0.951-1.013) | 0.241 | 0.733 (0.682-0.784) |
| GLS |  | 1.031 (0.978-1.087) | 0.251 |  |
| Age |  | 1.054 (1.030-1.078) | <.001 |  |
| Previous MI |  | 1.806 (1.244-2.621) | 0.002 |  |
| CKD |  | 1.682 (1.077-2.626) | 0.022 |  |
| Diabetes |  | 1.291 (0.854-1.952) | 0.226 |  |
| Heart failure |  | 2.587 (1.551-4.316) | 0.000 |  |
| Hypertension |  | 0.995 (0.677-1.463) | 0.980 |  |
| Male sex |  | 0.864 (0.548-1.362) | 0.530 |  |
|  |  |  |  |  |
|  |  |  |  |  |
| Subgroup: LVEF < 40 |  |  |  |  |
|  |  |  |  |  |
| Variable | n | HR (95% CI) | p-value | c-stat (95% CI) |
|  |  |  |  |  |
| Age | 121 | 1.026 (0.990-1.064) | 0.158 | 0.626 (0.535-0.718) |
| Previous MI |  | 0.600 (0.269-1.338) | 0.212 |  |
| CKD |  | 1.038 (0.520-2.073) | 0.916 |  |
| Diabetes |  | 1.692 (0.932-3.072) | 0.084 |  |
| Heart failure |  | 1.893 (0.817-4.385) | 0.137 |  |
| Hypertension |  | 1.882 (0.979-3.617) | 0.058 |  |
| Male sex |  | 1.177 (0.560-2.471) | 0.667 |  |
|  | | | | |
| LVEF | 121 | 0.987 (0.944-1.032) | 0.575 | 0.632 (0.543-0.722) |
| Age |  | 1.025 (0.989-1.063) | 0.177 |  |
| Previous MI |  | 0.583 (0.255-1.334) | 0.201 |  |
| CKD |  | 1.042 (0.519-2.092) | 0.909 |  |
| Diabetes |  | 1.648 (0.892-3.044) | 0.111 |  |
| Heart failure |  | 1.832 (0.787-4.262) | 0.160 |  |
| Hypertension |  | 1.853 (0.960-3.574) | 0.066 |  |
| Male sex |  | 1.175 (0.559-2.470) | 0.670 |  |
|  | | | | |
| GLS | 121 | 1.153 (1.051-1.264) | 0.003 | 0.696 (0.624-0.767) |
| Age |  | 1.019 (0.985-1.055) | 0.273 |  |
| Previous MI |  | 0.635 (0.277-1.455) | 0.283 |  |
| CKD |  | 0.856 (0.419-1.750) | 0.670 |  |
| Diabetes |  | 1.564 (0.863-2.836) | 0.140 |  |
| Heart failure |  | 2.351 (0.915-6.042) | 0.076 |  |
| Hypertension |  | 1.744 (0.907-3.352) | 0.095 |  |
| Male sex |  | 1.104 (0.507-2.405) | 0.803 |  |
|  | | | | |
| LVEF | 121 | 1.016 (0.968-1.067) | 0.521 | 0.690 (0.617-0.764) |
| GLS |  | 1.171 (1.058-1.296) | 0.002 |  |
| Age |  | 1.020 (0.986-1.055) | 0.259 |  |
| Previous MI |  | 0.677 (0.285-1.611) | 0.378 |  |
| CKD |  | 0.847 (0.418-1.720) | 0.647 |  |
| Diabetes |  | 1.589 (0.885-2.855) | 0.121 |  |
| Heart failure |  | 2.535 (1.023-6.278) | 0.044 |  |
| Hypertension |  | 1.737 (0.901-3.349) | 0.099 |  |
| Male sex |  | 1.097 (0.501-2.401) | 0.818 |  |
|  |  |  |  |  |
|  |  |  |  |  |
| Subgroup: LVEF ≥ 50 |  |  |  |  |
|  |  |  |  |  |
| Variable | n | HR (95% CI) | p-value | c-stat (95% CI) |
|  |  |  |  |  |
| Age | 609 | 1.057 (1.031-1.085) | <.001 | 0.753 (0.693-0.813) |
| Previous MI |  | 2.329 (1.490-3.639) | 0.000 |  |
| CKD |  | 1.348 (0.723-2.512) | 0.347 |  |
| Diabetes |  | 1.249 (0.736-2.122) | 0.410 |  |
| Heart failure |  | 3.097 (1.607-5.968) | 0.001 |  |
| Hypertension |  | 0.932 (0.582-1.492) | 0.768 |  |
| Male sex |  | 1.045 (0.623-1.754) | 0.867 |  |
|  | | | | |
| LVEF | 609 | 1.011 (0.969-1.055) | 0.606 | 0.756 (0.698-0.815) |
| Age |  | 1.057 (1.031-1.085) | <.001 |  |
| Previous MI |  | 2.333 (1.495-3.642) | 0.001 |  |
| CKD |  | 1.347 (0.723-2.509) | 0.348 |  |
| Diabetes |  | 1.240 (0.727-2.113) | 0.430 |  |
| Heart failure |  | 3.138 (1.609-6.121) | 0.001 |  |
| Hypertension |  | 0.930 (0.582-1.487) | 0.763 |  |
| Male sex |  | 1.036 (0.617-1.740) | 0.894 |  |
|  | | | | |
| GLS | 609 | 1.032 (0.975-1.093) | 0.274 | 0.754 (0.693-0.814) |
| Age |  | 1.055 (1.028-1.083) | <.001 |  |
| Previous MI |  | 2.287 (1.456-3.591) | 0.001 |  |
| CKD |  | 1.375 (0.744-2.541) | 0.310 |  |
| Diabetes |  | 1.259 (0.747-2.123) | 0.387 |  |
| Heart failure |  | 2.997 (1.584-5.669) | 0.001 |  |
| Hypertension |  | 0.925 (0.575-1.487) | 0.747 |  |
| Male sex |  | 1.023 (0.605-1.731) | 0.932 |  |
|  | | | | |
| LVEF | 609 | 1.019 (0.977-1.063) | 0.386 | 0.759 (0.697-0.818) |
| GLS |  | 1.042 (0.981-1.106) | 0.179 |  |
| Age |  | 1.054 (1.027-1.082) | <.001 |  |
| Previous MI |  | 2.274 (1.447-3.573) | 0.000 |  |
| CKD |  | 1.382 (0.748-2.551) | 0.302 |  |
| Diabetes |  | 1.252 (0.741-2.115) | 0.400 |  |
| Heart failure |  | 3.064 (1.606-5.844) | 0.001 |  |
| Hypertension |  | 0.919 (0.574-1.472) | 0.724 |  |
| Male sex |  | 1.000 (0.590-1.696) | 1.000 |  |

Supplemental table 3.

Title: Stepwise model prediction with alternative baseline parameters selected by assumed clinical significance

Supplemental table 3. Stepwise multivariable Cox proportional hazard models by addition of LVEF and GLS to the baseline model. Endpoint is combined all-cause death and heart failure re-hospitalization.

Baseline parameters: age, sex, NTproBNP, pathologic Q-wave, bundle branch block, infarction type (with NSTEMI assumed as the higher risk subtype)

| Model 1 |  | HR (95% CI) | p-value | | c-stat (95% CI) |
| --- | --- | --- | --- | --- | --- |
|  |  |  | |  |  |
| Age |  | 1.041 (1.020 - 1.062) | | 0.000 | 0.701 (0.665 - 0.755) |
| Sex |  | 0.900 (0.610 - 1.327) | | 0.595 |  |
| NTproBNP |  | 1.364 (1.212 - 1.533) | | <.001 |  |
| Pathologic Q-wave |  | 1.072 (0.604 - 1.900) | | 0.813 |  |
| Bundle branch block |  | 1.509 (0.896 - 2.543) | | 0.122 |  |
| NSTEMI |  | 1.291 (0.932 - 1.788) | | 0.125 |  |
|  |  |  | |  |  |
| Model 2 |  | HR (95% CI) | p-value | | c-stat (95% CI) |
|  |  |  | |  |  |
| LVEF |  | 0.964 (0.948-0.980) | | <.001 | 0.736 (0.692 - 0.780) |
| Age |  | 1.044 (1.022-1.065) | | <.001 |  |
| Sex |  | 1.025 (0.692-1.519) | | 0.900 |  |
| NTproBNP |  | 1.264 (1.126-1.419) | | <.001 |  |
| Pathologic Q-wave |  | 0.965 (0.530-1.755) | | 0.907 |  |
| Bundle branch block |  | 1.218 (0.680-2.180) | | 0.507 |  |
| NSTEMI |  | 1.311 (0.941-1.827) | | 0.109 |  |
|  |  |  | |  |  |
| Model 3 |  | HR (95% CI) | | p-value | c-stat (95% CI) |
|  |  |  | |  |  |
| GLS |  | 1.095 (1.046-1.147) | | <.0001 | 0.734 (0.691-0.778) |
| Age |  | 1.037 (1.016-1.058) | | 0.001 |  |
| Sex |  | 0.966 (0.651-1.432) | | 0.862 |  |
| NTproBNP |  | 1.246 (1.107-1.404) | | 0.001 |  |
| Pathologic Q-wave |  | 0.986 (0.542-1.793) | | 0.962 |  |
| Bundle branch block |  | 1.304 (0.739-2.300) | | 0.359 |  |
| NSTEMI |  | 1.340 (0.962-1.866) | | 0.083 |  |
|  |  |  | |  |  |
| Model 4 |  | HR (95% CI) | | p-value | c-stat (95% CI) |
|  |  |  | |  |  |
| LVEF |  | 0.972 (0.953-0.991) | | 0.005 | 0.739 (0.695-0.782) |
| GLS |  | 1.038 (0.982-1.096) | | 0.187 |  |
| Age |  | 1.041 (1.020-1.063) | | 0.001 |  |
| Sex |  | 1.027 (0.691-1.525) | | 0.897 |  |
| NTproBNP |  | 1.239 (1.101-1.395) | | 0.000 |  |
| Pathologic Q-wave |  | 0.956 (0.521-1.751) | | 0.883 |  |
| Bundle branch block |  | 1.200 (0.664-2.170) | | 0.545 |  |
| NSTEMI |  | 1.326 (0.950-1.850) | | 0.097 |  |

Supplemental table 4.

Title. Sex stratified baseline data.

Supplemental table 4: Comparison of baseline characteristics. Non-parametric scale variables were analyzed with Mann Whitney U test and categorical variables were analyzed with Chi2 statistics. * data from RIKS-HIA, N in inclusion group = 817.

| Baseline characteristics | Men (n = 725) | Women (n = 216) | | p-value |
| --- | --- | --- | --- | --- |
| Age. years. median (IQR) | 65 (57-71) | 67 (61-73) | 0.121 | |
| Echocardiography. median (IQR) |  |  |  | |
| LVEF | 54.0 (46.0 to 59.5) | 57.0 (49.0 to 63.8) | 0.002 | |
| GLS | -14.7 (-17.5 to -11.6) | -15.3 (-18.4 to -12.1) | 0.148 | |
| Diagnosis of ACS. n (%) |  |  |  | |
| STEMI | 343 (47.3) | 96 (44.4) | 0.444 | |
| NSTEMI | 335 (46.2) | 92 (42.6) | 0.336 | |
| Medical history. n (%) |  |  |  | |
| Hypertension* | 343 (47.3) | 115 (53.2) | 0.126 | |
| Diabetes mellitus* | 148 (20.4) | 51 (23.6) | 0.312 | |
| Atrial fibrillation* | 29 (4.0) | 12 (5.6) | 0.214 | |
| Heart failure* | 38 (5.2) | 11 (5.1) | 0.931 | |
| History of stroke* | 40 (5.5) | 20 (9.2) | 0.048 | |
| History of myocardial infarction* | 122 (16.8) | 31 (14.3) | 0.415 | |
| Chronic kidney disease (GFR <60ml/min) | 106 (14.6) | 41 (19.0) | 0.136 | |
